# Long-Term Predictors of Major Adverse Cerebrovascular and Cardiac Events After Successful Transradial Chronic Total Occlusion Recanalization: Five-Year Results of the TRACTOR Study

**DOI:** 10.3390/jpm16070380

**Published:** 2026-07-16

**Authors:** Tímea Szigethi, Dorottya Olajos, Levente Molnár, István Ferenc Édes, György Bárczi, Dávid Becker, László Gellér, Béla Merkely, Zoltán Ruzsa

**Affiliations:** 1 Department of Cardiology and Vascular Medicine, Heart and Vascular Center, Semmelweis University, 1122 Budapest, Hungary; 2 Department of Invasive Cardiology, Bács-Kiskun County Hospital, Eaching Hospital of the Albert Szent-Györgyi Medical University, 6000 Kecskemét, Hungary; 3 Department of Invasive Cardiology, Cardiology Center, University of Szeged, 6725 Szeged, Hungary

**Keywords:** chronic total occlusion, transradial access, PCI, MACCE, long-term outcomes, personalized medicine, risk stratification

## Abstract

**Background:** Transradial access has become a preferred strategy for chronic total occlusion (CTO) percutaneous coronary intervention (PCI) because of lower access site complication rates and increasing feasibility for complex CTO techniques using large-bore slender or sheathless systems. However, long-term outcomes after successful transradial CTO recanalization and their predictors remain incompletely defined. We aimed to identify long-term clinical and procedural predictors of major adverse cerebrovascular and cardiac events (MACCEs) after successful transradial CTO PCI. **Methods:** We performed a prospective dual-center cohort study including 227 consecutive patients who underwent successful transradial CTO PCI at two high-volume catheterization laboratories with dedicated CTO programs. A total of 405 CTO PCI procedures were screened; all femoral access cases were excluded and only transradial cases were eligible. Baseline clinical characteristics, left ventricular ejection fraction (LVEF), lesion complexity including J-CTO score, coronary disease extent, and procedural variables were prospectively collected and/or verified from institutional databases. The primary endpoint was MACCEs, defined as a composite of all-cause death, non-fatal myocardial infarction, target vessel revascularization, and stroke/transient ischemic attack. Event rates were estimated using Kaplan–Meier methods. Predictors were explored using Cox proportional hazards regression with clinically relevant covariates and procedural characteristics entered into multivariable models. **Results:** Among 227 patients with successful transradial CTO recanalization and complete 5-year follow-up among survivors, cumulative MACCEs and all-cause mortality were 44.0% and 21.5%, respectively. In multivariable Cox analysis, prior myocardial infarction, right coronary artery target vessel, and a higher number of implanted stents were independently associated with increased MACCE risk, whereas previous PCI and preserved LVEF (≥40%) were associated with lower MACCE risk. For all-cause mortality, preserved LVEF was independently protective, while right coronary artery target vessel intervention was associated with increased mortality risk; severe chronic kidney disease showed a significant univariable association and remained a strong signal after multivariable adjustment. **Conclusions:** After successful transradial CTO PCI, long-term MACCEs appear to be driven primarily by baseline comorbidity and coronary disease burden. No deaths were related to access site bleeding, and vascular access was not associated with fatal complications. These findings contribute to personalized cardiovascular medicine by identifying readily available clinical, anatomical, and procedural factors that enable individualized long-term risk stratification following successful transradial CTO recanalization. Integrating these predictors into post-procedural assessment may support tailored secondary prevention, follow-up strategies, and patient management according to individual risk profiles.

## 1. Introduction

The transradial approach for coronary angiography and intervention was introduced in the early 1990s, with seminal reports demonstrating feasibility for both balloon angioplasty and coronary stent implantation [1,2]. Since then, radial access has become the dominant access strategy for PCI in many centers, primarily due to a lower incidence of access site bleeding and vascular complications compared with femoral access [3]. The durability and safety of transradial PCI programs have also been supported by long-term institutional experience, including outcomes in ST-segment elevation myocardial infarction [4].

Chronic total occlusion (CTO) is reported to account for 15–25% of all patients referred for coronary angiography [5]. CTO PCI remains among the most technically challenging coronary interventions. Contemporary CTO practice relies on systematic lesion assessment, dedicated crossing technologies, and algorithm-based strategy selection [6,7]. Multiple studies have demonstrated that both clinical profile and lesion characteristics influence procedural success and complications, and systematic evidence confirms that CTO PCI outcomes are modulated by comorbidity and lesion complexity [8]. The Japan-CTO (J-CTO) score is widely used to grade angiographic difficulty and predict procedural performance, particularly guidewire crossing efficiency [9].

The clinical role of CTO PCI continues to be refined. Randomized trials comparing CTO PCI with optimal medical therapy have shown mixed effects on hard endpoints but support improvements in symptoms, exercise capacity, and/or selected functional measures, thereby informing patient selection for recanalization [10,11,12,13,14]. In parallel, equipment innovation and technique refinement—along with contemporary algorithms such as hybrid and “minimalistic hybrid” approaches—have improved procedural efficiency and broadened the spectrum of lesions suitable for percutaneous recanalization [6,7].

As operator expertise has matured, transradial CTO PCI has become increasingly feasible even for complex lesions [15,16,17,18]. Dedicated platforms such as slender sheaths and sheathless guiding systems facilitate complex PCI through radial arteries [16,17,18], while large registry data suggest that radial CTO PCI can be associated with favorable procedural outcomes and reduced bleeding compared with femoral access in experienced settings [19]. In addition, recent reports indicate that switching from proximal to distal radial access can be implemented for CTO recanalization when clinically appropriate, expanding the transradial toolkit and potentially preserving proximal radial patency [20]. Contemporary national experience further supports the feasibility and safety of transradial CTO recanalization in routine practice [21].

Despite substantial advances in chronic total occlusion (CTO) percutaneous coronary intervention (PCI), previous studies have largely focused on procedural success, peri-procedural complications, and short-term clinical outcomes. Although successful CTO recanalization has been associated with symptomatic improvement and favorable survival outcomes, evidence regarding the long-term predictors of major adverse cerebrovascular and cardiac events (MACCEs) following successful transradial CTO PCI remains limited. Moreover, the majority of existing CTO PCI studies have predominantly evaluated transfemoral interventions, while data specifically addressing the prognostic significance of the transradial approach are scarce.

Identification of factors associated with adverse long-term outcomes after successful transradial CTO recanalization is clinically relevant for optimizing patient selection, improving risk stratification, and guiding post-procedural management strategies. In the era of personalized cardiovascular medicine, integrating readily available clinical, anatomical, and procedural characteristics may enable more individualized estimation of long-term risk and facilitate tailored secondary prevention and follow-up strategies according to each patient’s risk profile. Such an approach may improve clinical decision-making beyond procedural success alone. Accordingly, the present study sought to evaluate the long-term independent predictors of MACCEs in patients undergoing successful transradial CTO recanalization and to identify prognostic factors that may contribute to more personalized post-procedural management.

## 2. Materials and Methods

### 2.1. Study Design and Population

We conducted a prospective dual-center cohort study including consecutive patients who underwent CTO PCI via transradial access at two high-volume tertiary centers with dedicated CTO programs between 2018 and 2019. A total of 405 CTO PCI procedures were screened. All femoral access cases were excluded, and only patients treated exclusively via transradial access were eligible for inclusion. The indication for CTO PCI was in accordance with contemporary myocardial revascularization guidelines, including refractory angina despite optimal medical therapy or documentation of a large ischemic area in the territory of the occluded vessel [19,22,23]. Patients were eligible if (1) the target lesion met CTO criteria and (2) the procedure was technically successful via a transradial approach. Baseline demographics, laboratory, echocardiographic, angiographic, and stent-related data were prospectively collected and verified from institutional records. Before discharge, radial artery patency was assessed clinically, and duplex ultrasound was performed when abnormalities were suspected. All surviving patients completed follow-up to 5 years through outpatient visits and/or direct contact. Written informed consent was obtained from all patients, and the institutional ethics committee approved the study.

### 2.2. Definitions

CTO was defined as a coronary occlusion with TIMI 0 flow and estimated occlusion duration ≥ 3 months based on clinical history and/or prior angiography. CTO complexity was assessed using standard angiographic features and summarized using the J-CTO score.

Technical success was defined as restoration of antegrade flow with residual stenosis < 30% in the treated segment and TIMI 3 flow, without in-hospital major adverse events requiring urgent reintervention.

Access site complications were recorded using standard definitions and categorized as clinically relevant vascular events, consistent with registry-based definitions [3].

### 2.3. Data Collection and Procedural Characteristics

Baseline demographics, cardiovascular risk factors, comorbidities (including diabetes mellitus and chronic kidney disease), LVEF, angiographic findings (including multivessel disease and CTO complexity), and procedural characteristics were recorded in institutional databases and verified from medical records. Procedural variables included use of a retrograde strategy, total implanted stent length, and periprocedural complications. Access site complications were adjudicated using standard criteria and classified as clinically relevant vascular events.

### 2.4. Endpoints and Follow-Up

The primary endpoint was MACCEs, defined as a composite of all-cause death, non-fatal myocardial infarction, target vessel revascularization, and stroke/transient ischemic attack.

Clinical follow-up was obtained from hospital records, scheduled outpatient assessments, and direct contact according to local practice. Complete 5-year follow-up was available for all surviving patients. Causes of death were adjudicated on the basis of the available clinical documentation.

### 2.5. Statistical Analysis

Associations between candidate predictors and outcomes were evaluated using Cox proportional hazards regression. Clinically relevant variables were pre-specified for multivariable modeling, including age, diabetes mellitus, chronic kidney disease, LVEF, J-CTO score, multivessel disease, target vessel territory, and procedural characteristics. Hazard ratios (HRs) are reported with 95% confidence intervals (CIs). Variables with sparse observations or structural redundancy within the selected successful cohort were interpreted cautiously and were not emphasized in the final narrative interpretation. The proportional hazards assumption was assessed using standard diagnostics. A two-sided *p* value < 0.05 was considered statistically significant.

## 3. Results

### 3.1. Baseline, Angiographic and Procedural Characteristics of Patients

A total of 227 patients with successful transradial CTO recanalization were included after exclusion of femoral access procedures from 405 screened CTO PCI cases. Complete 5-year follow-up was available for all surviving patients. The mean age was 64.8 ± 10.1 years, and 71.3% were male. Hypertension, dyslipidemia, and diabetes mellitus were the most prevalent cardiovascular risk factors. Most patients presented with stable angina (CCS class II–III), and preserved left ventricular systolic function was observed in more than half of the cohort. The right coronary artery was the most common CTO target vessel, severe calcification was frequent, and lesion complexity ranged from mild to severe according to the J-CTO score. Baseline clinical, angiographic, and procedural characteristics of the patients are summarized in Table 1. Periprocedural complications were infrequent. Radial access did not result in any in-hospital bleeding events leading to death.

### 3.2. Clinical Follow-Up and Multivariate Analysis

During 5-year follow-up, the cumulative incidence of MACCEs was 44.0%, with an all-cause mortality rate of 21.5%, highlighting the persistently high residual cardiovascular risk and emphasizes the overall vulnerability of this cohort despite successful CTO revascularization. Target vessel revascularization occurred relatively frequently, whereas non-fatal myocardial infarction and stroke/TIA were less common. The five-year clinical outcomes are summarized in Table 2.

**Figure 1:**
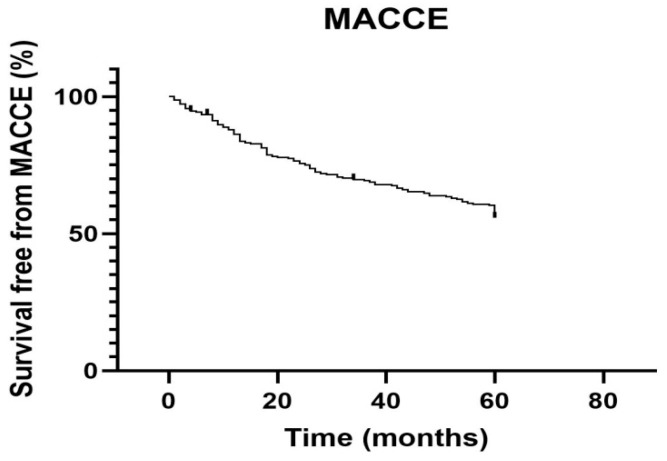
Kaplan–Meier curves for MACCEs.

Kaplan–Meier curves for MACCEs and survival are shown in Figure 1, demonstrating a progressive decline in event-free survival, with the steepest reduction occurring early after the index procedure.

In multivariable analysis, as summarized in Table 3 and Table 4, prior myocardial infarction, right coronary artery CTO, and a higher number of implanted stents were independently associated with increased MACCE risk, whereas previous PCI and preserved LVEF (≥40%) were associated with lower risk. For all-cause mortality, preserved LVEF remained independently protective, while right coronary artery CTO was associated with increased mortality risk. Severe renal dysfunction (GFR < 35 mL/min/1.73 m^2^) showed a significant association with mortality in univariable analysis and remained an important prognostic signal after multivariable adjustment. CTO-specific procedural variables were not independently associated with mortality.

## 4. Discussion

In this cohort of 227 consecutive patients undergoing successful transradial CTO PCI at two dedicated CTO centers, long-term adverse event rates remained substantial (MACCE 44.0%; mortality 21.5%) despite procedural success and systematic follow-up among survivors.

The principal findings are that late outcomes were associated predominantly with baseline clinical risk, target vessel territory, and procedural disease burden, whereas access-related complications were uncommon and did not appear to meaningfully influence long-term events.

### 4.1. Transradial CTO PCI and the Expanding Radial Toolbox

Transradial PCI has evolved from early feasibility reports for angioplasty and stenting [1,2] into a default access strategy in many catheterization laboratories, supported by lower rates of access site complications across PCI populations [3]. CTO PCI has simultaneously progressed through dedicated algorithms and devices, improving success rates and enabling recanalization of more complex occlusions [6,7].

In this context, transradial CTO PCI has become increasingly feasible, supported by slender and sheathless approaches that allow for complex guiding support through smaller vessels [15,16,17,18]. International registry data indicate that radial access for CTO PCI is associated with favorable procedural outcomes and bleeding advantages compared with femoral approaches, particularly in experienced programs [19]. Contemporary Hungarian experience also supports the feasibility of transradial CTO recanalization [21]. Importantly, distal radial access has emerged as an alternative puncturesite, andswitching between proximal and distal radial access may be a practical strategy for CTO recanalization in selected cases [20].

### 4.2. Drivers of Long-Term MACCEs: Comorbidity, Ventricular Function, and Complexity

Despite technically successful CTO recanalization, patients undergoing CTO PCI continue to exhibit a substantial residual cardiovascular risk attributable to advanced atherosclerotic disease and a high burden of comorbidities [23,24,25,26,27]. In the present study, Cox regression analysis identified baseline clinical characteristics, target vessel territory, and procedural disease burden as the principal determinants of long-term MACCEs following successful transradial CTO PCI. Prior myocardial infarction emerged as the strongest independent predictor of MACCEs, in agreement with previous studies demonstrating the association between prior infarction, impaired myocardial viability, adverse ventricular remodeling, and diffuse coronary atherosclerosis. Conversely, a history of previous PCI was independently associated with a lower incidence of adverse events, potentially reflecting more intensive implementation of secondary prevention measures and closer clinical follow-up, although residual confounding cannot be excluded. Among angiographic variables, right coronary artery CTO was independently associated with a higher risk of MACCEs compared with left anterior descending artery CTO, whereas a greater number of implanted stents remained independently associated with adverse outcomes, likely reflecting increased lesion complexity and overall coronary disease burden. Notably, neither J-CTO score, IVUS utilization, nor crossing strategy independently predicted late MACCEs. This observation is biologically plausible, given that the J-CTO score was originally developed to predict procedural complexity and guidewire crossing success rather than long-term clinical outcomes. In the mortality analysis, preserved left ventricular ejection fraction (LVEF ≥40%) was independently associated with lower mortality risk, whereas severe chronic kidney disease remained an important adverse prognostic factor after multivariable adjustment. Importantly, no procedural characteristics demonstrated an independent association with mortality, suggesting that patient-related and anatomical factors exert a greater influence on long-term prognosis than procedural variables in this population.

Our long-term results should be interpreted in the context of previously published CTO PCI studies and large contemporary registries, including the EuroCTO, PROGRESS-CTO, and Japanese CTO cohorts. Large registries have consistently demonstrated high procedural success rates with acceptable complication profiles, particularly in experienced centers applying contemporary CTO strategies. However, direct comparison between studies remains challenging because of differences in patient characteristics, lesion complexity, procedural approaches, and endpoint definitions.

In our cohort, the cumulative 5-year incidence of MACCEs was 44%, with all-cause mortality of 21.5%, non-fatal myocardial infarction of 2.2%, target vessel revascularization of 22.4%, and stroke/TIA of 2.6%. Although our use of some advanced CTO techniques differed from those reported in larger registries, our lesion characteristics did not suggest a uniformly low-complexity population, as reflected by a mean CTO length of 31.5 ± 3.6 mm, severe calcification in 79.2% of lesions, and 34.0% of patients belonging to the high-complexity J-CTO category (≥3). Therefore, the observed long-term outcomes are likely influenced by multiple factors beyond procedural strategy alone.

Collectively, these findings emphasize that successful CTO recanalization does not eliminate baseline cardiovascular risk, but rather represents an opportunity for symptomatic improvement and enhanced myocardial perfusion, while long-term prognosis remains strongly dependent on comprehensive secondary prevention, optimization of guideline-directed medical therapy, and careful longitudinal follow-up in high-risk patients.

### 4.3. Access SiteComplications and Long-Term Outcomes

A principal benefit of radial access is the reduction in access site and bleeding complications, particularly in the periprocedural period [3]. In our cohort, access site complications were infrequent and rarely contributed to late events, supporting the feasibility of a dedicated transradial CTO program in experienced centers; however, the absence of a femoral comparator precludes causal inference regarding access strategy and long-term outcome.

The exclusive use of the transradial approach represents an additional strength of our study. All femoral access cases were excluded from the screened CTO PCI population, thereby allowing for a focused assessment of long-term outcomes after successful radial CTO recanalization.

### 4.4. Lesion Territory and Coronary Complexity: Vessel-Specific Risk Signals

A novel observation in our MACCE modeling was the association between target vessel territory and long-term adverse events. Right coronary artery CTO was associated with a higher adjusted hazard of MACCEs compared with LAD CTO. This may relate to differences in coronary dominance, lesion morphology, treated segment length, distal runoff, or unmeasured disease burden; these mechanistic interpretations should be considered hypothesis-generating.

Several mechanisms may contribute. Right coronary artery CTOs may occur more often in patients with diffuse atherosclerotic burden, and the need for repeat revascularization, a major component of MACCEs in this cohort, may be more frequent in anatomically complex or long treated segments. Residual confounding remains possible because vessel territory may also act as a surrogate for unmeasured disease severity.

Notably, J-CTO score itself was not an independent long-term predictor of MACCEs in our models. This supports the concept that procedural complexity scores may be less informative for late outcomes once technical success has been achieved than global disease burden or clinical risk markers.

### 4.5. Procedural Surrogates: Stent Burden as a Marker of Diffuse Disease

Procedural complexity also appeared relevant. The number of implanted stents was independently associated with MACCEs and likely captures a combination of lesion length, diffuse disease, multiple treated segments, and overall atherosclerotic burden. In contrast, fluoroscopy time, radiation exposure, contrast use, and procedure duration were not independently associated with late outcomes.

TVR should be interpreted in relation to stent burden, which was substantial (~1.9 stents per lesion; ~49 mm total stent length), suggesting that repeat revascularization reflects lesion complexity and extensive stenting rather than procedural variability.

In contrast, other procedural factors, including procedural success (within an already successful cohort), use of intravascular ultrasound (IVUS), and procedural duration, were not independently associated with MACCEs. This may be explained by limited statistical power or the relatively homogeneous nature of a successfully treated population.

### 4.6. Clinical Implications

These findings suggest a pragmatic approach to post-CTO PCI care. Patients with reduced LVEF and/or advanced CKD represent a higher-risk phenotype in whom CTO PCI success should be followed by intensified guideline-directed therapy, strict riskfactor control, and close clinical follow-up. Greater stent burden may help identify patients at higher risk for future TVR-driven MACCEs.

From the perspective of personalized cardiovascular medicine, our findings demonstrate that long-term prognosis after successful transradial CTO PCI is influenced by a combination of readily available clinical, anatomical, and procedural characteristics rather than by procedural success alone. Previous myocardial infarction, left ventricular systolic function, target vessel location, and procedural complexity may help identify patients with distinct long-term risk profiles. Integrating these routinely available variables into post-procedural risk assessment may facilitate individualized follow-up intensity, optimization of secondary prevention, and more informed clinical decision-making. Although these findings require external validation before implementation in formal risk prediction models, they provide additional evidence supporting a personalized approach to long-term management after successful CTO recanalization.

### 4.7. Limitations

This study has limitations. First, the dual-center non-randomized design introduces potential selection bias and limits generalizability. Second, the analysis was restricted to successful transradial CTO PCI procedures; therefore, the findings should not be extrapolated to unsuccessful procedures or femoral access CTO PCI. Third, several advanced techniques such as IVUS guidance, retrograde crossing, and dissectionre-entry were used infrequently, limiting statistical power for these variables. Fourth, the multivariable models should be interpreted with appropriate caution because some candidate predictors were sparse and residual confounding is possible. Finally, potentially relevant follow-up variables, including medication adherence, serial lipid levels, frailty, and detailed lesion morphology, were not captured and may have contributed to residual confounding. The ACEF score is a validated and widely used predictor of outcomes in CTO PCI, integrating key clinical determinants of risk. Although not assessed in the present study, it remains a relevant tool for risk stratification and emphasizes the importance of baseline clinical risk in interpreting procedural and long-term outcomes.

## 5. Conclusions

Preserved ventricular function predicts better long-term outcomes after successful transradial CTO PCI, whereas prior myocardial infarction, right coronary artery target vessel intervention, and greater stent burden identify patients at higher risk of long-term MACCE. Overall, patient-related and disease-burden factors appear to be more important determinants of late outcome than access-related factors or most procedural variables in this selected successful transradial cohort.

A personalized, risk- and anatomy-guided approach may optimize outcomes in CTO PCI by integrating clinical and lesion-specific characteristics.

Our findings contribute to personalized cardiovascular medicine by demonstrating that routinely available clinical, anatomical, and procedural factors can identify patients at different long-term risk after successful transradial CTO PCI. Integrating these predictors into clinical practice may improve individualized risk stratification, support tailored secondary prevention, and optimize follow-up strategies.

## Figures and Tables

**Table 1:** Baseline clinical, angiographic, and procedural characteristics.

Characteristic	Overall (*N* = 227)
**Demographics**	
Age, years	64.8 ± 10.1
Male sex, n (%)	162 (71.3%)
Body mass index, kg/m^2^	29.2 ± 4.9
**Cardiovascular risk factors**	
Hypertension, n (%)	207 (91.1%)
Diabetes mellitus, n (%)	105 (46.2%)
Dyslipidemia, n (%)	179 (78.8%)
Current smoking, n (%)	45 (19.8%)
**Medical history**	
Prior myocardial infarction, n (%)	89 (39.2%)
Prior PCI, n (%)	92 (40.5%)
Prior CABG, n (%)	26 (11.4%)
Chronic kidney disease, n (%) #	29 (12.7%)
**Clinical status/imaging**	
Clinical presentation (stable angina), n (%)	221 (97.3%)
Clinical presentation (ACS), n (%)	6 (2.6%)
LVEF, % - Normal (50–70%) - Decreased (30–50%) - Low (<30%)	113 (58.6%) 75 (33%) 14 (6,1%)
Reduced LVEF (<40%), n (%)	32 (14%)
**Angiographic and lesion characteristics**	
Target vessel (RCA), n (%)	108 (47.5%)
Target vessel (LAD), n (%)	69 (30.3%)
Target vessel (LCx), n (%)	39 (17.8%)
Multivessel disease, n (%)	81 (35.8%)
CTO length, mm	31.5 ± 3.6
Proximal cap ambiguity, n (%)	98 (43.1%)
Severe calcification, n (%)	180 (79.2%)
Tortuosity, n (%)	46 (20.2%)
Prior failed CTO attempt, n (%)	24 (10.5%)
J-CTO score	
J-CTO category (0–1), n (%)	35 (12.6%)
J-CTO category (2), n (%)	147 (53.2%)
J-CTO category (≥3), n (%)	94 (34.0%)
**Procedural characteristics**	
Primary access (radial), n (%)	227 (100%)
Bilateral radial access, n (%)	88 (38.7%)
Sheath size (6F), n (%)	214 (94.2%)
Sheath size (7F/slender), n (%)	6 (2.6%)
Sheathless guiding, n (%)	19 (8.4%)
Retrograde strategy used, n (%)	11 (4.8%)
Dissection/re-entry used, n (%)	4 (1.7%)
Procedure time, min	45.7 (41–49)
Fluoroscopy time, min Radiation dose (DAP)	21.9 (19–24) 1854
Contrast volume, mL	158 (147–168)
Number of stents per procedure	1.9 ± 1.2
Total stent length, mm	48.6 (44–52)
Intravascular imaging (IVUS/OCT), n (%)	11/1 (4.8/0.4%)
**Periprocedural complications**	
Coronary perforation, n (%)	11 (4.8%)
Pericardial tamponade, n (%)	2 (0.8%)
Donor vessel complication, n (%)	3 (1.3%)
Periprocedural MI, n (%)	4 (1.8%)
Major bleeding, n (%)	0 (0%)
Access site complication *, n (%)	12 (5.2%)

Notes: # Chronic kidney disease was defined as KDIGO stage G3b or worse; the source manuscript specifies GFR 30–44 mL/min/1.73 m^2^. * Access site complications included clinically relevant vascular events such as BARC ≥ 2 bleeding, large hematoma, pseudoaneurysm, arteriovenous fistula, or radial artery occlusion requiring treatment.

**Table 2:** Five-year clinical outcomes.

Outcome	Events (n)	5-Year Cumulative Incidence (%)
MACCE (primary composite)	100	44%
All-cause death	49	21.5%
Non-fatal myocardial infarction	5	2.2%
Target vessel revascularization	51	22.4%
Stroke/TIA	6	2.6%

**Table 3:** Cox regression analysis for MACCEs.

Predictor	Univariable HR (95% CI)	*p*-Value	Multivariable HR (95% CI)	*p*-Value
**Clinical factors**				
Male	0.35 (0.15–0.81)	0.0147	0.13 (0.04–0.42)	0.00055
Hypertension	0.32 (0.09–1.08)	0.0662	0.26 (0.05–1.40)	0.118
Previous PCI	0.49 (0.21–1.14)	0.0979	0.34 (0.13–0.92)	0.0336
Dyslipidemia	0.47 (0.17–1.27)	0.138	0.57 (0.14–2.38)	0.442
BMI	1.10 (0.94–1.29)	0.232	Not entered	--
Previous MI	1.50 (0.66–3.42)	0.337	3.46 (1.19–10.08)	0.0225
Family history	0.68 (0.25–1.83)	0.446	0.97 (0.31–3.03)	0.964
Diabetes	0.72 (0.29–1.74)	0.460	0.81 (0.25–2.61)	0.721
GFR <35	0.73 (0.25–2.16)	0.575	0.26 (0.04–1.48)	0.128
Age (per 1 year)	0.99 (0.95–1.03)	0.665	0.98 (0.94–1.03)	0.515
Smoking	1.14 (0.42–3.09)	0.789	3.54 (0.72–17.47)	0.121
Previous CABG	0.94 (0.22–4.00)	0.928	1.58 (0.24–10.53)	0.636
**TTE parameters**				
EF ≥40% (vs. <40%)	0.48 (0.27–0.85)	0.0120	0.53 (0.29–0.95)	0.0338
Significant valvular disease (yes vs. no)	1.91 (1.07–3.40)	0.0284	1.70 (0.94–3.07)	0.0795
**Anatomical factors**				
J-CTO score (per +1)	0.87 (0.68–1.12)	0.295	0.88 (0.68–1.15)	0.358
Target CX vs. LAD	1.41 (0.88–2.26)	0.156	A	A
Target RCA vs. LAD	1.31 (0.91–1.89)	0.149	1.63 (1.05–2.52)	0.0289
Target LM vs. LAD	0.46 (0.11–1.86)	0.276	A	A
**Procedural factors**				
IVUS guidance	0.63 (0.26–1.55)	0.318	0.59 (0.24–1.46)	0.257
Anterograde vs. retrograde strategy	1.51 (0.81–2.81)	0.194	1.57 (0.84–2.94)	0.158
Stent number (+1)	1.19 (1.02–1.38)	0.0246	1.35 (1.02–1.78)	0.0335
Stent length (+10 mm)	1.04 (0.98–1.11)	0.168	0.95 (0.85–1.06)	0.332
Contrast volume (+100 mL)	0.91 (0.73–1.13)	0.397	0.78 (0.56–1.10)	0.158
Radiation (DAP +1000)	0.98 (0.91–1.06)	0.601	0.99 (0.91–1.07)	0.751
Fluoro time (+10 min)	1.00 (0.95–1.06)	0.920	1.01 (0.91–1.11)	0.873
Procedure time (+10 min)	1.01 (0.91–1.12)	0.858	1.08 (0.90–1.29)	0.431

**Table 4:** Cox regression analysis for death.

Predictor	Univariable HR (95% CI)	*p*-Value	Multivariable HR (95% CI)	*p*-Value
**Clinical factors**				
Male	0.93 (0.52–1.65)	0.798	0.83 (0.45–1.56)	0.573
Hypertension	0.72 (0.31–1.68)	0.451	0.76 (0.30–1.94)	0.563
Dyslipidemia	0.71 (0.40–1.27)	0.252	0.79 (0.41–1.51)	0.475
GFR <35	2.06 (1.11–3.83)	0.022	1.75 (0.92–3.36)	0.090
Diabetes	1.22 (0.73–2.05)	0.453	1.32 (0.77–2.26)	0.319
Smoking	0.95 (0.49–1.84)	0.888	0.93 (0.46–1.86)	0.830
Family history	0.53 (0.21–1.32)	0.170	0.57 (0.22–1.47)	0.248
Previous MI	1.21 (0.72–2.03)	0.480	1.53 (0.86–2.72)	0.152
Previous PCI	0.67 (0.39–1.15)	0.144	0.58 (0.32–1.05)	0.0706
Previous CABG	0.50 (0.16–1.61)	0.245	0.47 (0.14–1.54)	0.210
**TTE parameters**				
EF ≥40% (vs. <40%)	0.32 (0.16–0.63)	0.0010	0.41 (0.19–0.87)	0.0197
Significant valvular disease	2.51 (1.19–5.30)	0.016	1.65 (0.73–3.72)	0.230
**Anatomical factors**				
J-CTO score (per +1)	1.16 (0.81–1.66)	0.422	1.18 (0.81–1.71)	0.382
Target CX vs. LAD	0.96 (0.46–2.03)	0.920	1.54 (0.63–3.74)	0.341
Target RCA vs. LAD	1.66 (0.98–2.82)	0.060	2.01 (1.05–3.82)	0.0346
Target LM vs. LAD	1.10 (0.27–4.53)	0.891	1.64 (0.37–7.35)	0.519
**Procedural factors**				
IVUS guidance	1.14 (0.41–3.16)	0.796	0.99 (0.35–2.78)	0.981
ADR vs. AWE strategy	1.36 (0.49–3.76)	0.552	1.36 (0.48–3.87)	0.560
Stent number (+1)	0.94 (0.75–1.18)	0.612	1.27 (0.83–1.94)	0.268
Stent length (+10 mm)	0.95 (0.88–1.04)	0.287	0.88 (0.75–1.04)	0.137
Contrast (+50 mL)	0.87 (0.73–1.03)	0.103	0.78 (0.60–1.02)	0.066
DAP (+1000)	1.00 (0.90–1.10)	0.986	1.06 (0.96–1.18)	0.273
Procedure time (+10 min)	0.95 (0.88–1.04)	0.265	0.96 (0.84–1.11)	0.624
Fluoroscopy time (+10 min)	0.95 (0.81–1.12)	0.541	1.13 (0.88–1.46)	0.337

## Data Availability

The data presented in this study are available on request from the corresponding author (the data are not publicly available due to privacy or ethical restrictions).

## Abbreviations

The following abbreviations are used in this manuscript:

AWE	antegrade wire escalation
ADR	antegrade dissection re-entry
CABG	coronary artery bypass graft surgery
CKD	chronic kidney disease
CTO	chronic total occlusion
CX/LCx	left circumflex artery
Fr	French
J-CTO	Japan CTO score
LAD	left anterior descending artery
LM	left main coronary artery
LVEF	left ventricular ejection fraction
PCI	percutaneous coronary intervention
RCA	right coronary artery
RWE	retrograde wire escalation
RDR	retrograde dissection re-entry
